# Slope-Reducing High Tibial Osteotomy and Over-The-Top Anterior Cruciate Ligament Reconstruction With Achilles Tendon Allograft in Multiple Failed Anterior Cruciate Ligament Reconstruction

**DOI:** 10.1016/j.eats.2022.07.019

**Published:** 2022-10-20

**Authors:** Bálint Zsidai, Emre Anil Özbek, Ian D. Engler, Janina Kaarre, Ehab M. Nazzal, Andrew J. Curley, Volker Musahl

**Affiliations:** a Department of Orthopaedic Surgery, UPMC Freddie Fu Sports Medicine Center, University of Pittsburgh, Pittsburgh, USA; b Department of Orthopaedics, Institute of Clinical Sciences, Sahlgrenska Academy, University of Gothenburg, Gothenburg, Sweden; c Department of Orthopedics and Traumatology, İbn'i Sina Training and Research Hospital, Ankara University, Ankara, Turkey

## Abstract

Graft failure is a challenging complication following anterior cruciate ligament reconstruction (ACL-R). Among the multiple anatomic and nonanatomic risk factors contributing to ACL-R failure, there is accumulating evidence that a posterior tibial slope of 12° or greater may predispose patients to graft failure of primary and revision ACL-R. In addition, previously malpositioned or widened tunnels, as well as limited autograft options, pose challenges in the setting of revision ACL-R. This Technical Note describes a technique to correct an increased posterior tibial slope using slope-reducing high tibial osteotomy, and single stage revision ACL-R with Achilles tendon allograft using the over-the-top route, in a single-stage procedure. The surgical technique involves an anterior approach to the proximal tibia, followed by tibial tubercle osteotomy and anterior closing-wedge osteotomy. The posterior cortical osteotomy hinge is left intact below the insertion of the posterior cruciate ligament. Over-the-top revision ACL-R is then performed using an Achilles tendon allograft passed around the posterior aspect of the lateral femoral condyle and fixed onto the lateral femur.

Revision anterior cruciate ligament reconstruction (ACL-R) after multiple graft failures requires careful evaluation of the factors underlying repeated failure, as well as strategies to correct them. Posterior tibial slope (PTS) is a major contributor to recurrent instability and graft failure in patients undergoing ACL-R.^1^^,^^2^ Recent biomechanical studies have demonstrated the detrimental effect of increased PTS on anterior tibial translation (ATT) and forces transmitted through the native and reconstructed anterior cruciate ligament (ACL).^3^^,^^4^ Clinical studies further illustrate the negative impact of increased PTS on the integrity of the reconstructed ACL, with a PTS of 12° or greater suggested in the literature as a strong predictor of repeat ACL injury, especially in the young, active population.^1^^,^^2^

Current described techniques for slope-reducing high tibial osteotomy (HTO)^5^, ^6^, ^7^ aim to correct disadvantageous knee kinematics in patients with increased PTS and lead to improved knee function in patients undergoing concurrent revision ACL-R. Performing a slope-reducing HTO may be indicated in the setting of a revision ACL-R, in patients with a PTS >12°, and neutral or only slightly varus tibiofemoral alignment.^5^

Limited graft options, malpositioned and widened tunnels, and hardware removal present hurdles in the multiple revision setting, which often require staged surgical procedures to overcome.^8^ The over-the-top (OTT) technique for ACL-R^9^ provides the advantage of bypassing the limitations of revision surgery. By shuttling the graft around the lateral femoral condyle, previously misplaced femoral tunnels and associated hardware may be disregarded. Moreover, the possibility of both allograft and autograft use renders the OTT technique a versatile approach for revision ACL-R.

In patients undergoing multiple revision ACL-R, an aggregation of factors often leads to persistent instability and technically demanding surgical scenarios. Slope-reducing HTO and OTT revision ACL-R may be performed concurrently to mitigate recurrent instability caused by an increased PTS and the previously described challenges inherent to multiple revision ACL-R.

This article presents a single-stage technique for revision ACL-R in the setting of multiple graft failures, combining transtubercle slope-reducing HTO and OTT Achilles tendon allograft ACL-R (Video 1).

## Surgical Technique (With Video Illustration)

### Preoperative Evaluation and Planning

**Fig 1:**
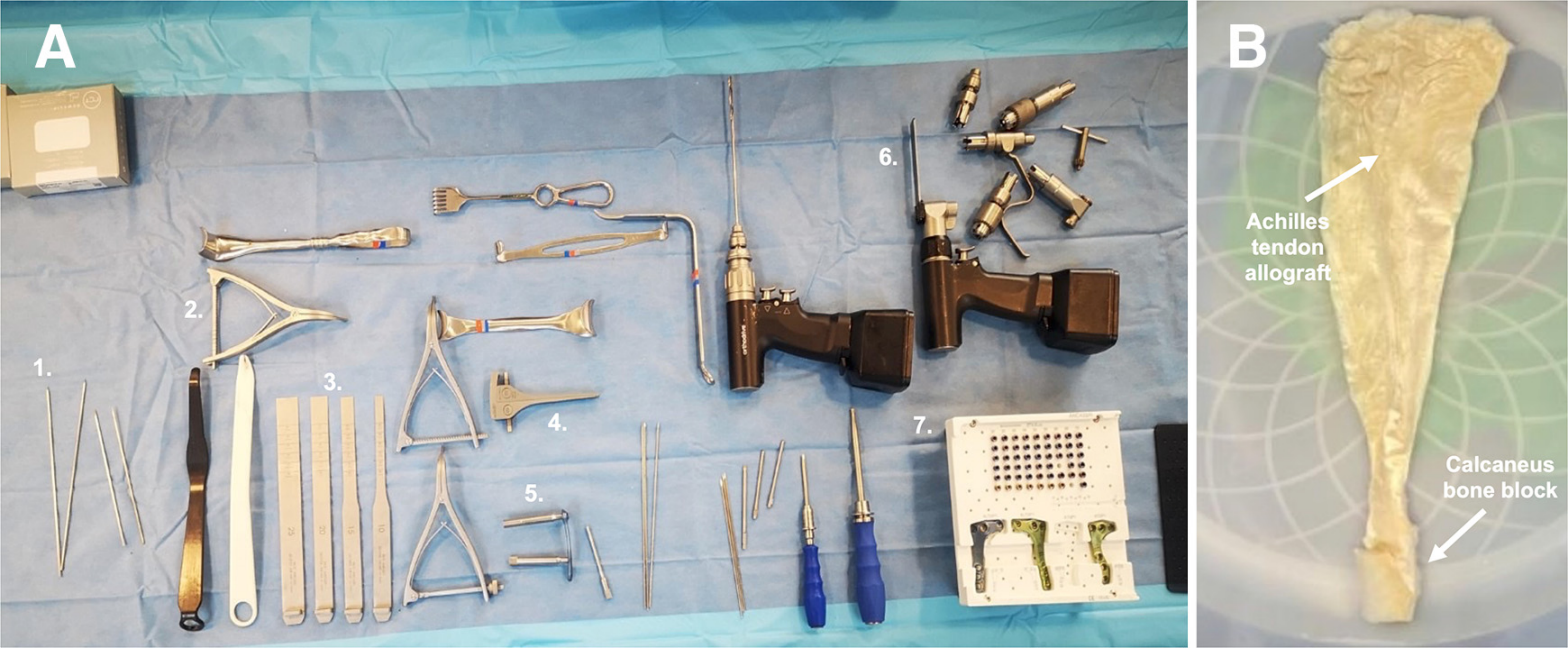
(A) Surgical equipment used for slope-reducing high tibial osteotomy. Guide pins (1.), lamina spreader (2.), osteotomes (3.) osteotomy distractor (4.), drill sleeves (5.), oscillating saw (6.), and high tibial osteotomy plates with locking screws (7.) are displayed. (B) The Achilles tendon allograft with an associated 11-mm diameter and 20-mm length calcaneus bone block (arrows) is sized to a length of 15 cm and a diameter of 11 mm for over-the-top anterior cruciate ligament reconstruction.

Lower-extremity alignment, previous tibial and femoral tunnels, and existing hardware associated with previous ACL-Rs are evaluated using strict lateral and long-cassette radiographs. The assessment of PTS on the lateral radiograph is performed using the concentric circles technique, by measuring the angle between the anatomic tibial axis and a line tangential to the tibial plateau^10^ (Fig 1A). The magnitude of slope correction is then calculated to yield a PTS of 7 to 10°, which is considered the mean range in the literature.^11^ Re-rupture of the ACL graft and concomitant meniscus, cartilage, and ligament pathology are evaluated using magnetic resonance imaging. Computed tomography scan with 3-dimensional reformatting is the best study to evaluate previous tunnel position and size.

### Patient Positioning and Intraoperative Examination

The patient is positioned supine on the operating room table. After induction of general anesthesia, the left lower extremity is prepped and draped in standard sterile fashion. A well-padded pneumatic tourniquet is placed around the thigh. Examination under anesthesia includes comparison of knee range of motion and Lachman, anterior drawer, and pivot shift tests between the surgical and non-surgical knees. An L-bar and side post are placed to maintain 90° of flexion of the surgical knee.

### Slope-Reducing HTO

**Fig 2:**
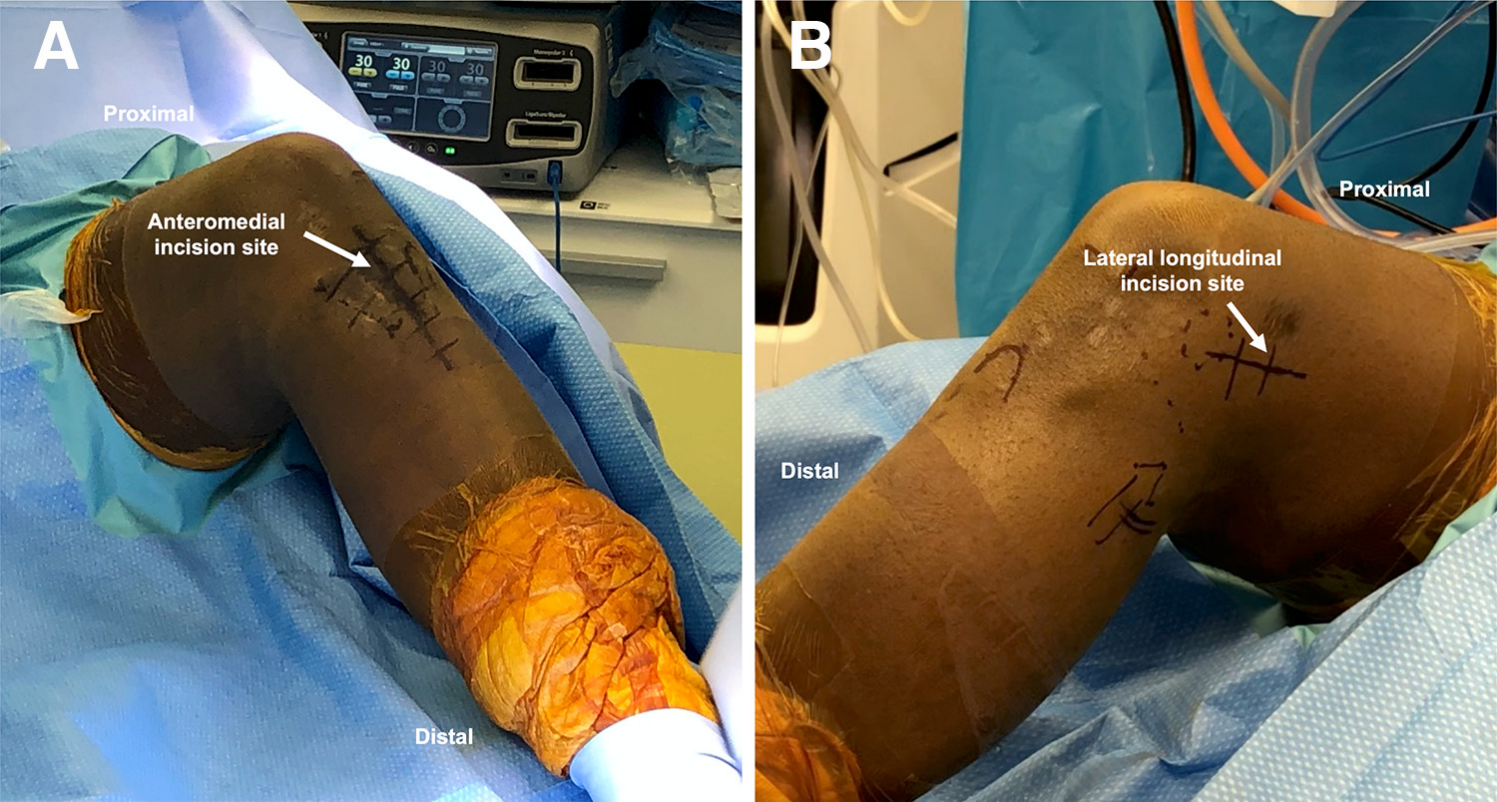
(A) The anteromedial incision (arrow) used for approach to the proximal tibia and tibial tubercle is marked and extended along the previous surgical incision. (B) The lateral longitudinal incision (arrow) used to access the over-the-top position is marked along the posterolateral aspect of the lateral femoral condyle.

**Fig 3:**
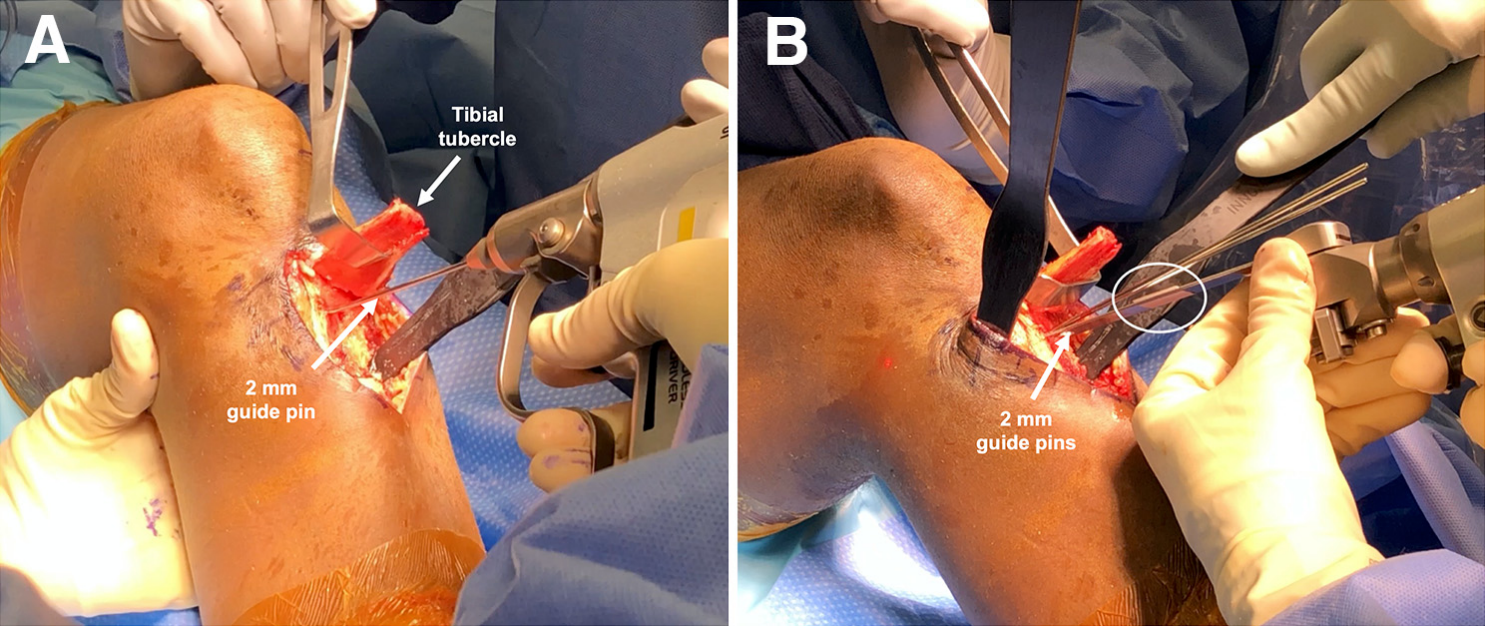
(A) Two 2-mm guide pins are placed in the proximal tibia, located 5 cm distal to the anterior joint line and 7 mm apart proximal-to-distal. The pins are aimed toward the posterior aspect of the tibial plateau, just distal to the footprint of the posterior cruciate ligament, and advanced just into the posterior tibial cortex. The tibial tubercle (arrow) is everted. (B) The sawblade is marked using adhesive tape (oval) according to the distance traversed by the tibial guide pins to avoid injury to neurovascular structures in proximity of the posterior tibia. An oscillating saw is used to perform 2 anteroposterior bone cuts. The second bone cut is made 7 mm distal to the site of the first.

An anteromedial incision (Fig 2A) is performed in line with the previous surgical incision if able, extending approximately 7 cm distal to the tibial tubercle. The patellar tendon is identified and protected during the approach to the tibial tubercle. Tibial tubercle osteotomy is performed using an oscillating saw to obtain a 1-cm thick and 5-cm long bone block (Fig 3A).^5^ The tibial tubercle is everted proximally.

**Fig 4:**
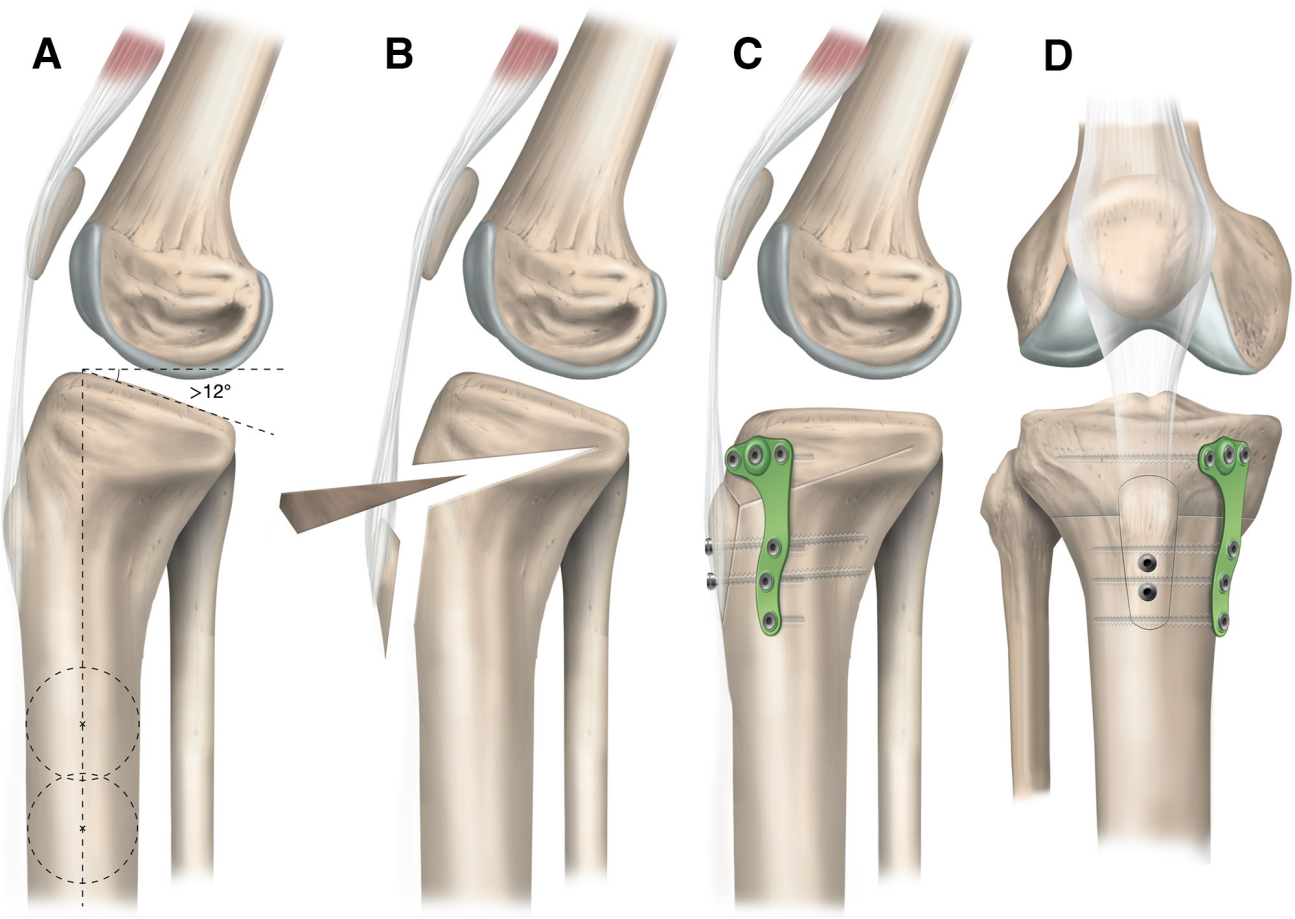
Schematic illustration of slope-reducing high tibial osteotomy. (A) A posterior tibial slope >12 measured using lateral radiography in the setting of failed anterior cruciate ligament reconstruction is an indication for slope-reducing high tibial osteotomy. (B) The tibial tubercle is osteotomized and everted proximally to provide access to the proximal tibia at the level of the tibial tubercle, where subsequent resection of a bony wedge is performed to achieve slope reduction. (C) The osteotomy line is reduced and fixated using an osteotomy plate on the anteromedial aspect of the proximal tibia, followed by shortening, reduction, and fixation of the tibial tubercle using two 4.5-mm cortical screws. (D) The anterior aspect of the final construct demonstrates the final position of the tibial tubercle and fixation of the osteotomy plate with locking screws.

Location of the transtubercle HTO is determined under fluoroscopic guidance with the knee in 90° of flexion, using convergent placement of two 2-mm guide pins in the proximal tibia, with the proximal pin placed approximately 5 cm distal to the anterior joint line (Fig 3). As an example, for a 7° slope correction, the 2 pins are spaced 7 mm apart on the anterior tibial cortex, and aimed towards the posterior aspect of the tibial plateau just distal to the footprint of the posterior cruciate ligament. The pins are advanced just into the posterior tibial cortex. To avoid damage to the popliteal neurovascular structures and posterior cortex of the tibia, which serves as the hinge point of the slope-reducing osteotomy, the length of the guide pins in the tibia is marked on the sawblade of the oscillating saw used during subsequent steps with adhesive tape (Fig 3B).^5^^,^^12^ The oscillating saw is used to make 2 anteroposterior bone cuts along the guide pins, 7 mm apart proximal-to-distal, with care taken not to violate the posterior tibial cortex. The saw depth is checked frequently under fluoroscopy and is advanced shy of the tape on the blade. The last centimeter of the osteotomy may be performed with an osteotome for greater control. Large osteotomes are used to facilitate resection of the bony wedge interposed between the osteotomy sites (Fig 1). The integrity of the osteotomy hinge at the posterior tibial cortex is confirmed using fluoroscopy, and the osteotomy gap is gradually reduced under full knee extension and application of manual pressure, achieving a slope reduction of approximately 7°. The magnitude of slope correction and potential overcorrection leading to genu recurvatum are assessed using fluoroscopy. Medial osteosynthesis is performed using an HTO plate (Newclip Technics, Haute-Goulaine, France). Temporary fixation of the osteotomy plate on the medial aspect of the tibia is achieved using 4.0-mm guide pins inserted in the corresponding polyaxial and monoaxial plate holes above and below the osteotomy cut (Fig 1C). Final fixation of the osteotomy plate is achieved by the insertion of 4.5-mm locking screws. The tibial tubercle bone block is shortened by 7 mm, corresponding to the anterior length of the osteotomized wedge, and reduced onto the anterior tibial cortex. Following temporary fixation with two guide pins, the tibial tubercle is secured using two 4.5-mm cortical screws using lag-by-technique. In the event of poor bone quality, a 6.5-mm cancellous screw may be used instead (Fig 4A).

### Over-the-Top ACL-R

**Fig 5:**
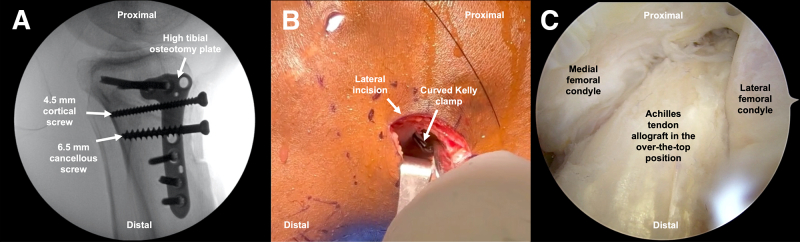
(A) The tibial tubercle is secured using lag-by-technique with a 4.5-mm cortical screw proximally and a 6.5 mmm cancellous screw distally (arrows) due to poor bone quality. (B) Lateral aspect of the knee displaying a curved Kelly clamp inserted through the anteromedial portal and advanced through the posterolateral capsule into the over-the-top position. (C) Arthroscopic image displaying the course of the Achilles tendon allograft in the over-the-top position, shuttled around the lateral femoral condyle.

An Achilles tendon allograft with associated 11-mm diameter and 20-mm length bone block (Fig 5B) is prepared and sized to a tendinous length of 15 cm and a diameter of 11 mm. Sutures are passed through drill holes in the bone block. The tendinous end of the graft is whipstitched with No. 2 braided suture. Diagnostic knee arthroscopy is performed using standard anterolateral and anteromedial (AM) portals. The previous ACL graft is debrided, allowing direct visualization of the associated femoral tunnel and the OTT position.

A lateral longitudinal incision (Fig 2B) is performed along the posterolateral aspect of the lateral femoral condyle, and dissection is extended toward the OTT position on the posterior aspect of the lateral femoral condyle,^9^ followed by incision of the posterior third of the iliotibial band. The lateral femoral condyle ACL insertion and OTT position are rasped to create a surface for graft healing. A curved Kelly clamp is inserted through the AM portal and advanced through the posterolateral capsule in the OTT position (Fig 4B). The clamp is advanced into the lateral incision, where it is palpated and then visualized. The looped end of a shuttling suture is fed into the clamp, which is withdrawn, bringing the suture loop through the AM portal. The clamp may be difficult to open, but repetitive firm attempts to open it alongside pushing the clamp in and out will dilate the capsule sufficiently to permit opening the clamp.

**Fig 6:**
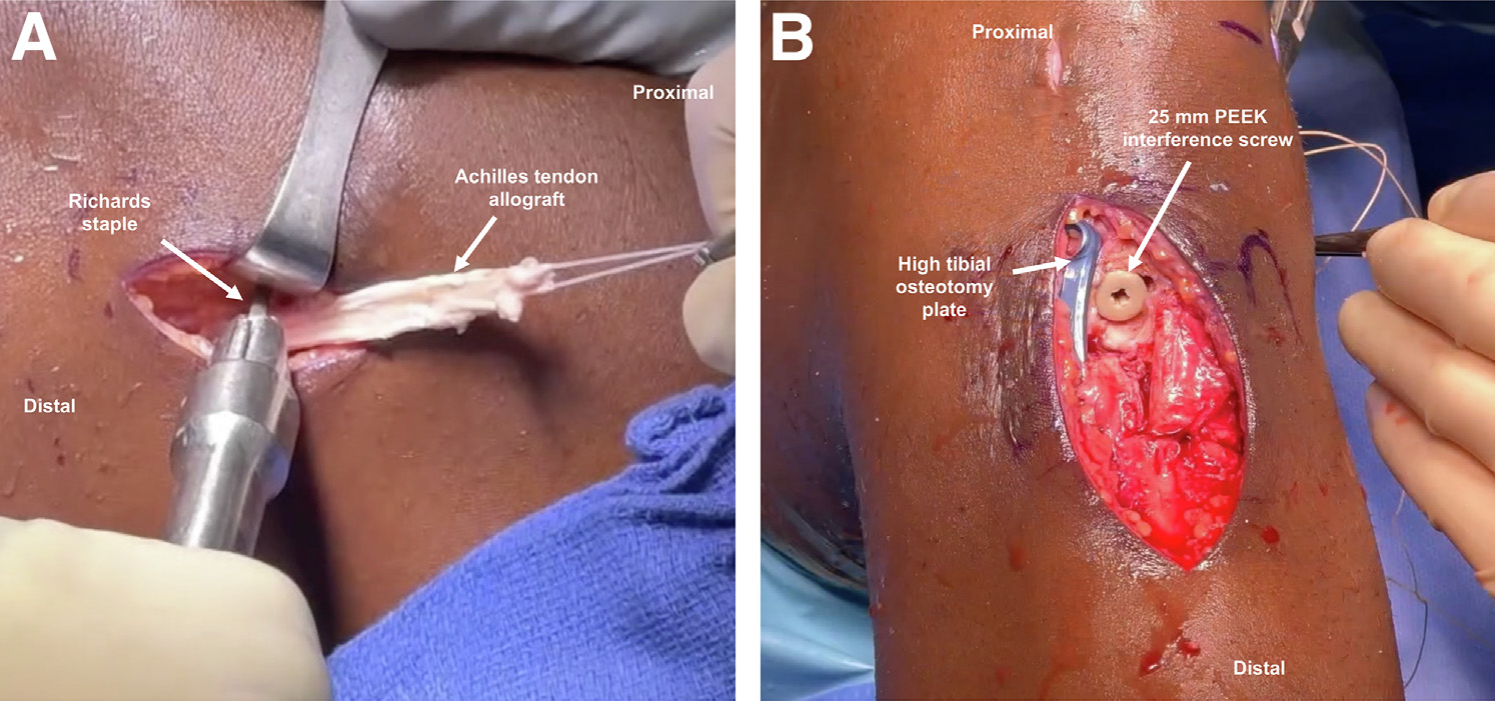
(A) Lateral aspect of the knee displaying fixation of the Achilles tendon allograft posterior to the lateral femoral epicondyle using two small Richards staples (arrows). (B) Anteromedial view demonstrating tibial fixation of the Achilles tendon allograft bone block using a 25-mm PEEK interference screw and high tibial osteotomy plate (arrows).

An ACL tip-aimer guide set at 55° is introduced via the AM portal with the bullet on the anteromedial tibial cortex, and a guide pin is placed. If the previous tibial tunnel is anatomic, then it may be used in this step. Otherwise, a different guide pin trajectory ensures that the tunnels do not collapse into each other. If the guide pin lifts a screw from the HTO plate, then a different starting point and trajectory should be used. The guide pin is reamed over with an 11-mm reamer. The shuttling suture is retrieved through the tibial tunnel using a suture grasper. The shuttling suture passes the sutures of the tendinous end of the Achilles tendon allograft from the anterior tibia, through the OTT position (Fig 4C), and out of the lateral thigh incision. The graft is then advanced until the bone block is flush with the tibial cortex. Femoral fixation of the soft-tissue portion of the allograft just posterior to the lateral femoral epicondyle is achieved using 2 small Richards staples (Fig 6A). Staples should be placed perpendicular to the surface of the bone and should not pierce the graft.

While placing tension on the tibial-sided sutures, the graft is cycled through 10 repetitions of flexion and extension for stress relaxation. The knee is then positioned in 10° of flexion with a posterior drawer, and tibial fixation (Fig 6B) using a 25-mm PEEK (polyether ether ketone) interference screw (BIOSURE PK screw, 11 mm × 25 mm; Smith & Nephew, Andover, MA) is performed. Adequate position and tension of the graft is evaluated under arthroscopy, ensuring full range of motion and the absence of graft impingement. Following closure of the surgical site, a sterile surgical dressing and knee brace are placed on the lower extremity.

Of note, this technique may be combined with a lateral extra-articular tenodesis (LET) by using a longer 25-cm Achilles allograft.^13^ Following routing of the graft around the lateral femoral condyle, it is brought distally out of an incision 1 cm medial to the Gerdy tubercle. Fixation is performed with 2 staples on the lateral femoral condyle followed by one staple on the anterolateral tibia. Addition of an LET has been shown to increase rotatory knee stability,^14^ which may be helpful in the patient with multiple ACL graft failures.

### Postoperative Rehabilitation

Patients are restricted to non–weight-bearing during the first 4 weeks following surgery, with transition to partial weight-bearing from weeks 6 to 8 to prevent injury to the osteotomy site. To prevent stiffness, active and passive knee range of motion exercises may be performed as tolerated in the immediate postoperative period. Once weight-bearing begins at week 6, progressive rehabilitation consists of partial weight-bearing with closed-chain exercises, with a focus on regaining full range of motion and strength, followed by light-impact aerobic exercises.

## Discussion

Slope-reducing osteotomy, as shown in this study, is a salvage procedure and can be combined with single-stage revision ACL-R. The OTT technique is a simple and anatomic technique that provides the advantage of circumventing previous femoral tunnel/hardware and eliminates the potential for repeat femoral tunnel non-anatomic malposition during revision ACL-R (Table 1). In the setting of failed ACL-R, addressing the causes underlying graft re-rupture and residual instability is paramount. Previously misplaced tunnels, concomitant meniscal tears, and increased PTS are all potential contributors to ACL-R failure and should be addressed using the appropriate surgical techniques.^8^

**Table 1:** Pearls and Pitfalls of Slope-Reducing HTO and Over-The-Top Revision ACL-R With Achilles Tendon Allograft

Pearls	Pitfalls
• Over-the-top revision ACL-R may be advantageous in patients with overlapping or widened previous femoral tunnels	• The targeted range of PTS is 7-10°. Overcorrection may lead to genu recurvatum and an increase in forces transmitted through the posterior cruciate ligament.
• Patient-specific instrumentation may be helpful during preoperative planning and intraoperatively to ensure accurate slope-reduction and to plan tibial tunnel placement.	• Fracture of the osteotomy hinge and injury of the popliteal artery are serious complications, which can be avoided with fluoroscopic guidance, careful use of the oscillating saw, and finalizing the osteotomy with osteotomes
• Removal of a 1-mm thick bony wedge corresponds to approximately 1° of PTS reduction • The posterior cortex of the tibia should be protected by marking the length of the inserted tibial guide pins on the blade of the oscillating saw.	• Resection of the distal aspect of the tibial tubercle should be performed in a manner that permits reduction of the tubercle after completion of the slope-reducing osteotomy.
• To reduce the osteotomy gap, gradual compression is provided by the chest of the assistant surgeon against the sole of the foot.	• Avoid excessive anterior placement of the osteotomy plate, which may obstruct the optimal position of the tibial tunnel.
• Eccentric screw placement in the compression plates provides additional compression at the osteotomy site.	• The surgeon should be aware of complications of slope-reducing HTO, including wound infection, coronal plane malalignment, compartment syndrome, and nonunion
	• Inappropriately placed staples can pierce or cut the allograft and provide inadequate fixation

ACL-R, anterior cruciate ligament reconstruction; HTO, high tibial osteotomy; PTS, posterior tibial slope.

Biomechanical studies have documented the negative effect of increased PTS on ATT and the forces transmitted through the native and reconstructed ACL.^3^^,^^4^ Reduction of PTS reduces ATT and ACL graft forces, which explains the benefits of slope-reducing HTO in ACL-reconstructed knees with an increased PTS.^4^^,^^15^ Recent clinical studies demonstrate the correlation between increased PTS and ACL-R failure, reporting up to an 11-fold increased risk of graft failure, especially in the young, athletic population.^1^^,^^2^ Currently, slope-reducing HTO may be indicated in the setting of a revision ACL-R, in patients with a PTS >12°, and neutral to only slightly varus lower extremity alignment. Contraindications include genu recurvatum >10°, varus deformity >5°, and advanced knee chondrosis.^5^

Several techniques for slope-reducing HTO are described in the literature,^5^, ^6^, ^7^ with the major difference being the location of the osteotomy cut in relation to the tibial tubercle. Good-to-excellent objective and subjective knee functional outcomes have been achieved using each of the existing approaches.^6^^,^^7^^,^^10^^,^^16^ In the presently described technique, HTO is performed at the level of the tibial tubercle (Fig 1).^5^ It is proposed that HTO performed above the level of the tibial tubercle should be avoided in patients with patella alta^17^ and may increase the risk of injury to the patellar tendon.

Compared with primary ACL-R, revision ACL-R, and especially multiple revision ACL-R, have increased rates of graft failure and inferior outcomes.^18^ The challenges of tunnel management in revision ACL-R lead some surgeons to choose a 2-stage procedure with bone grafting. Unfortunately, 2-stage ACL-R exposes the patient to the risks of an additional surgical procedure and extends recovery time. The OTT technique bypasses widened or malpositioned femoral tunnels, providing a versatile and cost-effective approach to revision ACL-R. An important additional benefit of the OTT technique is the anatomic nature of the graft due to the posterior course taken around the lateral femoral condyle, given that many failed ACL-Rs have femoral tunnels that are placed too anterior.^19^

Although allograft use in the setting of revision ACL-R is controversial, autograft options may be limited in a multiple revision setting. Studies investigating differences in the risk of failure when comparing autograft and allograft revision ACL-R are inconclusive, and there is no clear advantage of autograft tissue in the revision setting outside of the young, active population.^20^^,^^21^ Consequently, allograft ACL-R using the OTT technique should be considered as an alternative in patients with lower activity demands.

Graft failure following revision ACL-R using the OTT technique has been reported to be 8.4%, with a 52% rate of return to pre-injury activity levels.^22^ Moreover, the OTT route combined with an LET restores rotatory knee laxity in both patients who are skeletally immature and patients undergoing revision ACL-R^23^ and yields clinical outcomes comparable to single- and double-bundle revision ACL-R.^24^

In conclusion, this Technical Note describes a surgical technique for revision ACL-R, combining slope-reducing HTO and over-the-top ACL-R with Achilles tendon allograft to address both the factors underlying graft failure and the challenges inherent to multiple revision ACL-R. Slope-reducing HTO eliminates the negative impact of increased PTS on the ACL graft and restores anteroposterior stability to the knee joint. Additionally, the OTT technique provides a versatile surgical approach by circumventing challenges encountered in the revision setting, such as malpositioned or widened tunnels, presence of femoral hardware, and decreased options for autograft harvest.

## Supplementary Data

ICMJE author disclosure forms

Video 1 This video demonstrates a single-stage technique for slope-reducing high tibial osteotomy (HTO) and over-the-top (OTT) ACL reconstruction with Achilles tendon allograft in a multiple revision setting. Intraoperative physical examination includes Lachman, anterior drawer, and pivot shift tests. Surgery begins with a tibial tubercle osteotomy and a transtubercle HTO. The prior anteromedial tibial incision is used and dissection is taken down to the periosteum. The patellar tendon is identified and protected. Bovie cautery is used to define the anteromedial aspect of the tibial tubercle osteotomy and subperiosteal dissection is taken along the posteromedial border of the tibia, as well as the lateral aspect of the tibia. An oscillating saw is used to create the tibial tubercle osteotomy, including an inferior transverse cut. The tibial tubercle is freed and everted proximally. A guide pin is placed 5 cm distal to the joint line, aiming just posterior to the posterior cruciate ligament insertion on the posterior tibia. The correct position is confirmed under fluoroscopy. A second guide pin is placed 7 mm distal to the first, aiming for a convergent point on the posterior tibia. An oscillating saw is used to make 2 osteotomy cuts parallel to each pin, creating a wedge of bone in the anterior tibia. Osteotomes may be used to complete the osteotomy and to remove the anterior wedge of bone. A radiograph shows the tibia following removal of the bony wedge. The osteotomy site is debrided and reduced with extension of the tibia. The HTO plate is placed over the anteromedial tibia and is held in place with guide pins. Locking screws are placed throughout the plate for permanent fixation. Radiographs show the osteotomy following reduction and fixation. The tibial tubercle is then reduced and fixated with two screws. Attention is turned to the over-the-top anterior cruciate ligament (ACL) reconstruction. Incision is taken through the skin and the posterior third of the iliotibial band, down to the OTT position at the posterior aspect of the lateral femoral condyle. A “high and tight” anterolateral arthroscopy portal is made, and diagnostic arthroscopy is performed. Following debridement of the prior ACL graft, a curved Kelly clamp is passed into the OTT position through the anteromedial portal. The curved Kelly clamp can be palpated and seen, and is used to shuttle a suture from the anteromedial portal through the anterolateral incision. Tibial tunnel drilling proceeds in standard fashion with a tip-aimer guide, guide pin, and reamer. A suture is passed and the allograft is shuttled through the tibial tunnel into the OTT position. The Achilles tendon allograft in an anatomic position is visualized with arthroscopy. Tension is placed on the tibial sutures and the Achilles tendon allograft is fixed on the lateral femur, just posterior to the lateral epicondyle, with 2 Richards staples. The graft is cycled 10 times with tension on the tibial sutures. With a posterior drawer, and tension on the tibial sutures at 10° of flexion, an interference screw is placed. Postfixation examination shows no pivot shift. Postoperative radiographs are displayed.
